# Consensus Gene Network Analysis Identifies the Key Similarities and Differences in Endothelial and Epithelial Cell Dynamics after *Candida albicans* Infection

**DOI:** 10.3390/ijms241411748

**Published:** 2023-07-21

**Authors:** Surabhi Naik, Akram Mohammed

**Affiliations:** 1 Department of Surgery, College of Medicine, University of Tennessee Health Science Center, Memphis, TN 38103, USA; 2 Center for Biomedical Informatics, College of Medicine, University of Tennessee Health Science Center, Memphis, TN 38103, USA

**Keywords:** host–pathogen, correlation networks, RNA-sequence data, immune response, *Candida albicans*, endothelial cells, epithelial cells

## Abstract

Endothelial and epithelial cells are morphologically different and play a critical role in host defense during *Candida albicans* infection. Both cells respond to *C. albicans* infection by activating various signaling pathways and gene expression patterns. Their interactions with these pathogens can have beneficial and detrimental effects, and a better understanding of these interactions can help guide the development of new therapies for *C. albicans* infection. To identify the differences and similarities between human endothelial and oral epithelial cell transcriptomics during *C. albicans* infection, we performed consensus WGCNA on 32 RNA-seq samples by relating the consensus modules to endothelial-specific modules and analyzing the genes connected. This analysis resulted in the identification of 14 distinct modules. We demonstrated that the magenta module correlates significantly with *C. albicans* infection in each dataset. In addition, we found that the blue and cyan modules in the two datasets had opposite correlation coefficients with a *C. albicans* infection. However, the correlation coefficients and *p*-values between the two datasets were slightly different. Functional analyses of the hub of genes from endothelial cells elucidated the enrichment in TNF, AGE-RAGE, MAPK, and NF-κB signaling. On the other hand, glycolysis, pyruvate metabolism, amino acid, fructose, mannose, and vitamin B6 metabolism were enriched in epithelial cells. However, mitophagy, necroptosis, apoptotic processes, and hypoxia were enriched in both endothelial and epithelial cells. Protein–protein interaction analysis using STRING and CytoHubba revealed *STAT3*, *SNRPE*, *BIRC2*, and *NFKB2* as endothelial hub genes, while *RRS1*, *SURF6*, *HK2*, and *LDHA* genes were identified in epithelial cells. Understanding these similarities and differences may provide new insights into the pathogenesis of *C. albicans* infections and the development of new therapeutic targets and interventional strategies.

## 1. Introduction

*Candida albicans* is an opportunistic pathogen in the human body, usually residing in the gastrointestinal tract, mouth, and vaginal area [1,2,3,4]. However, when the balance of microorganisms in the body is disturbed, *C. albicans* can overgrow and cause infections ranging from superficial skin infections to life-threatening systemic infections [5,6,7,8]. Endothelial and epithelial cells play important roles in the response to these infections and act as barriers to prevent *C. albicans* invasion [9,10]. When *Candida* breaches these barriers, endothelial and epithelial cells initiate a series of cellular and molecular responses to clear the infection and prevent its dissemination [11]. Endothelial cells, which line the inner surface of blood vessels, are the first cells encountered by circulating *Candida* [12]. They play a crucial role in recruiting immune cells to the site of infection. Endothelial cells respond to *C. albicans* infection by expressing adhesion molecules, such as intercellular adhesion molecule-1 (ICAM-1) and vascular cell adhesion molecule-1 (VCAM-1), which promote the binding and migration of leukocytes to the site of infection [13,14]. In addition, endothelial cells can directly kill *C. albicans* by producing nitric oxide and reactive oxygen species [15]. While much is known about the clinical manifestations of *C. albicans* infections, our understanding of the underlying molecular mechanisms remains limited.

Epithelial cells, which line the surfaces of the skin, respiratory tract, gastrointestinal tract, and genitourinary tract, provide a physical barrier against *C. albicans* invasion [16] and secrete antimicrobial peptides [17], such as defensins and cathelicidins, which can directly kill *C. albicans* [18,19]. In addition, both endothelial and epithelial cells produce cytokines and chemokines that recruit immune cells to the site of infection and stimulate the activation of antifungal immune responses [20,21]. Hence, the responses of endothelial and epithelial cells to these infections are critical for the initiation and coordination of immune responses required for the effective clearance of the pathogen. However, in certain circumstances, *C. albicans* can evade host defenses, leading to persistent infections that can be difficult to treat.

Solid organ transplant (SOT) patients are at increased risk of developing *C. albicans* infections as these patients often have weakened immune systems because of the use of immunosuppressive drugs, use of antibiotics, and comorbidities [22,23,24]. Therefore, endothelial and epithelial cells are important for controlling these infections.

In recent years, there has been a growing interest in understanding the molecular mechanisms underlying host–pathogen interactions. Among the various approaches to deciphering these complex interactions, consensus gene network analysis has emerged as a powerful tool for identifying key genes and pathways that are involved in cellular responses to infection. Recently, we reported genes from human endothelial cells in response to *C. albicans* infection across two different computational methods (WGCNA and DEG) [25]. Here, we developed a novel approach to study the cell dynamics of human endothelial and epithelial cells during *C. albicans* infection to elucidate the common and unique pathways between these cell types. In this work, we applied the consensus WGCNA to analyze 32 RNA seq samples from in vitro *C. albicans* infection in human endothelial and oral epithelial cells after infection and controls. Additionally, we identified central players in each module, which could be novel targets to investigate epithelial and endothelial cells during *C. albicans* infection. We performed a consensus WGCNA analysis between human endothelial and epithelial cell samples, identified 14 related gene modules, and derived the functional analysis of epithelial cells from the corresponding human endothelial cell modules. We identified a number of genes and pathways that were differentially regulated in endothelial and epithelial cells following *C. albicans* infection. Interestingly, we also found a core set of genes and pathways that were commonly regulated in both cell types, suggesting that there are fundamental similarities in the host response to *C. albicans* infection across different cell types.

These findings have important implications for our understanding of the molecular mechanisms that underlie the host response to *C. albicans* infection. By identifying key genes and pathways that are commonly regulated in different cell types, this study provides a more comprehensive picture of the host response to *C. albicans* infection than was previously possible. At the same time, the identification of cell-type-specific differences in gene expression profiles provides important clues for the development of targeted therapies and diagnostic tools for *C. albicans* infections, as well as for our broader understanding of host–pathogen interactions. In addition to its immediate clinical relevance, this study also has broader implications for our understanding of host–pathogen interactions more generally. By applying a powerful computational technique to integrate data from multiple experiments, we have demonstrated the power of a systems biology approach to unraveling the complex molecular interactions that underlie infectious diseases.

## 2. Results

### 2.1. Identification of Consensus Gene Modules between Endothelial and Epithelial Cells

**Figure 1:**
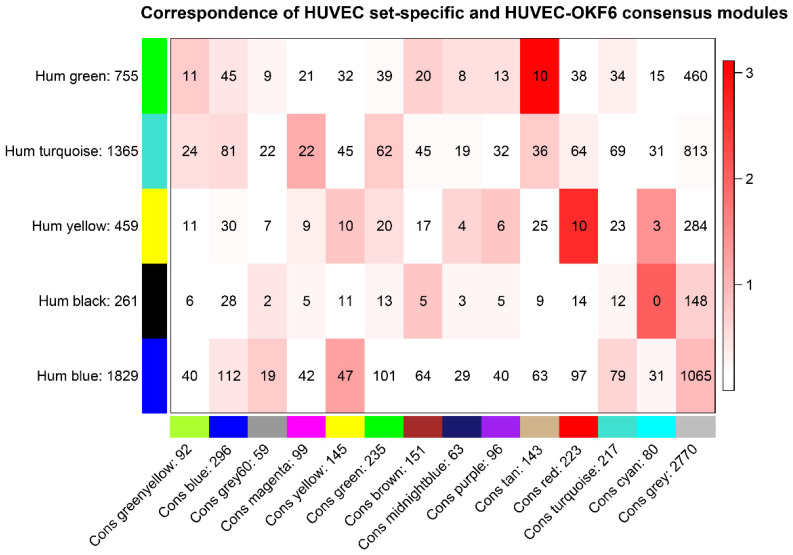
Correspondence between the human endothelial and consensus (endothelial/epithelial) modules. The rows show human endothelial modules and the columns show the consensus modules of endothelial and epithelial cells.

To identify the differences and similarities between endothelial and oral epithelial transcriptomics during *C. albicans* infection, we performed consensus WGCNA and analyzed the connected genes. We clustered consensus module eigengenes (MEs) based on consensus correlation across endothelial and epithelial samples. Then, we merged the modules whose expression profiles were very similar (Supplementary Figure S1), resulting in 14 modules. Figure 1 shows that the green and yellow modules of endothelial cells associate with their corresponding tan and red consensus modules, which indicates that the module structure in epithelial cells is somewhat similar to the endothelial cell expression data. The numbers represent the shared gene counts and show that some endothelial modules have a consensus counterpart. The intensity of the red color shows −log(p), which demonstrates the degree of overlap.

### 2.2. Module Association with External Trait

**Figure 2:**
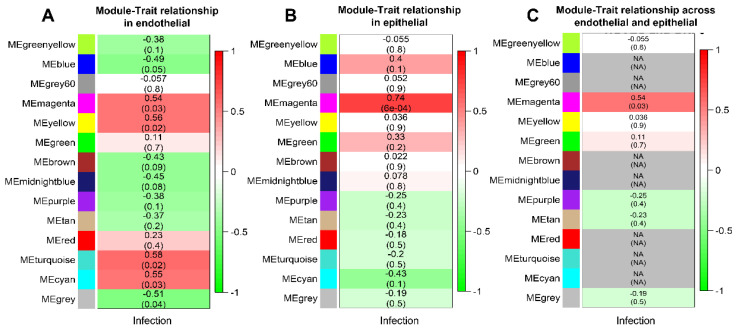
Consensus module construction between endothelial (HUVEC) and epithelial (OKF6) cell types. Pearson correlation coefficients between infection status and module eigengenes in (**A**) endothelial cells and (**B**) epithelial cells. (**C**) Pearson correlation coefficients between infection status and consensus module eigengenes. Numbers in brackets indicate the corresponding *p*-values.

We also determined the relationship between consensus modules and external traits (infection status) in endothelial and epithelial cells. The module–trait relationship in endothelial cells showed that the magenta, yellow, turquoise, cyan, blue, and grey modules are associated with infection status (Figure 2A). The epithelial module trait relationship showed that only the magenta module correlated with infection status (Figure 2B). Therefore, we demonstrate that the magenta module significantly correlates with infection in each cell type. However, the correlation coefficients and *p*-values between the two cell types differed. When the correlation coefficient values for endothelial and epithelial cell types for a specific module had the same sign, we took the lowest value as the correlation coefficient of the comparison module (Figure 2C). However, when the two correlation coefficients had opposite signs, we set the correlation coefficients of the comparison module to NA (not available). Notably, we found that the blue and cyan modules in the two cell types had opposite correlation coefficients with infection status.

### 2.3. Differential Consensus Eigengene Network Analysis Reveals a Preserved Network Structure

**Figure 3:**
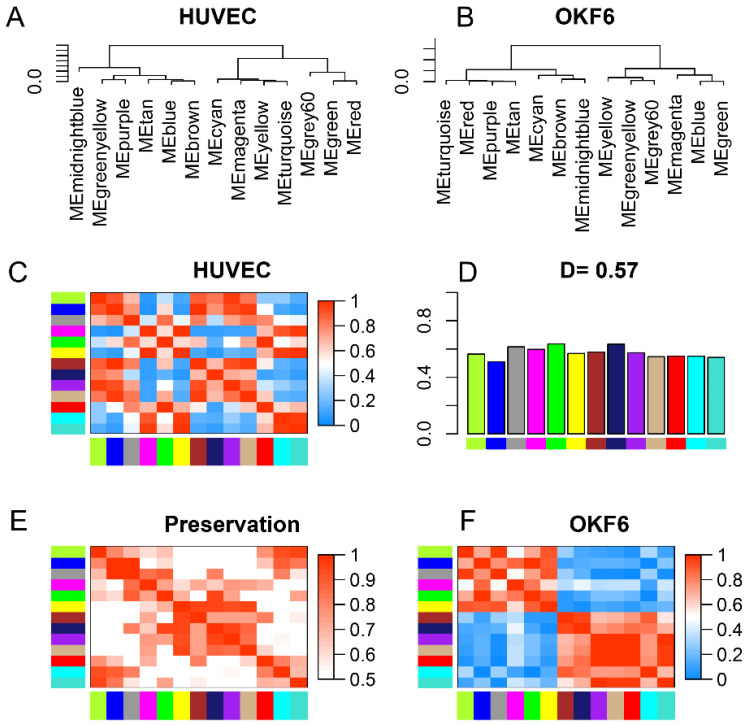
Consensus eigengene networks and their differential analysis. (**A**,**B**) Dendrograms of the consensus MEs in the endothelial and epithelial cell datasets. (**C**,**F**) Eigengene network heatmaps. The red color represents high adjacency (i.e., positive correlation) and the blue represents low adjacency. (**D**) Mean preservation of adjacency for each eigengene to all other eigengenes. (**E**) Preservation network heatmap.

Further, we performed a differential analysis of consensus eigengene networks in endothelial and epithelial cell samples (Figure 3) to identify correlation preservation. Figure 3A,B demonstrate eigengene dendrograms of endothelial and epithelial cells, respectively. Figure 3C,F display heatmaps of the eigengene network. The endothelial and epithelial cell network heatmap illustrates that the inter-module relationships in these two datasets are different. Figure 3D exhibits mean network preservation for each eigengene, i.e., the column means of the preservation heatmap. The overall preservation of the two networks achieved a moderate value of 0.57, showing both similarities and differences in the correlated network structure. Figure 3E represents the pair-wise preservation adjacency of the eigengene network of endothelial and epithelial cells, i.e., one minus the absolute difference of the eigengene networks in endothelial and epithelial cells. The value of preservation defines the correlation preservation between pairs of module eigengenes across the two networks. Though the preservation shown by red clusters represents the correlated modules, these could possibly include genes that differ within the network.

### 2.4. Functional Enrichment Analyses for Correlated Consensus Modules from Endothelial and Epithelial Cells

**Figure 4:**
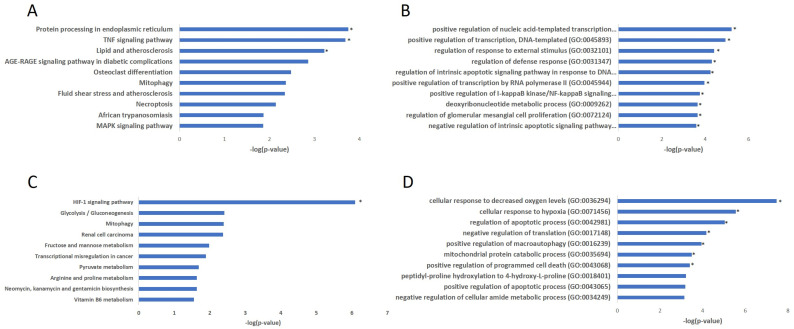
KEGG and GO enrichment analysis for the magenta consensus module genes. (**A**,**B**) Endothelial cells; (**C**,**D**) epithelial cells. The top 10 enriched terms for the input gene set from endothelial and epithelial cells are displayed based on the −log10(*p*-value). An asterisk (*) indicates the term also has a significant adjusted *p*-value (<0.05).

To understand the biological role of the highly correlated consensus module (magenta) with infection status (Figure 2C), we performed KEGG and Gene Ontology (GO) analyses to identify the biological pathways that are significantly enriched (FDR *p* < 0.05) for endothelial and epithelial cells. For endothelial cells, KEGG analyses elucidated that the magenta module is enriched in TNF signaling, AGE-RAGE signaling, MAPK signaling pathways, and mitophagy (Figure 4A). For endothelial cells, Gene Ontology analyses revealed that the genes involved in the magenta module are highly enriched in defense response, response to external stimuli, positive regulation of NF-κB signaling, and apoptotic signaling pathway (Figure 4B). For epithelial cells, the magenta module is enriched in HIF-1 signaling, mitophagy, renal cell carcinoma, glucose, vitamin, pyruvate, and antibiotic metabolism (Figure 4C). For the epithelial cells, Gene Ontology analyses revealed that the magenta module is enriched in hypoxia, cell death, apoptotic process, and macroautophagy (Figure 4D). Overall, our enrichment analysis showed that the topmost enriched pathways in endothelial cells were TNF (tumor necrosis factor) signaling, AGE-RAGE signaling, NF-κB signaling, MAPK signaling, lipid, and atherosclerosis. While in epithelial cells, the enriched pathways were glycolysis, pyruvate metabolism, amino acid, fructose, mannose, and vitamin B6 metabolism. Meanwhile, mitophagy, necroptosis, and apoptotic processes, as well as hypoxia and HIF-1 signaling, were common in endothelial and epithelial cells.

**Figure 5:**
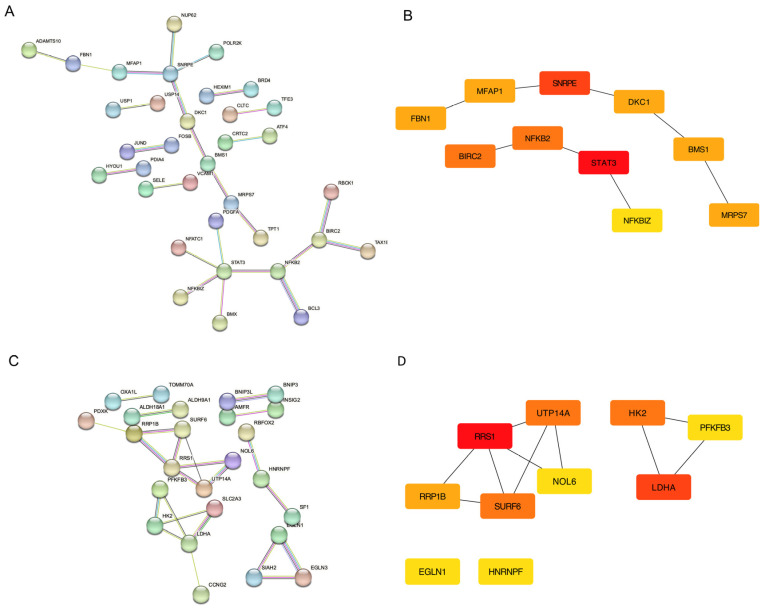
Protein–protein interaction network of the magenta module in (**A**) human endothelial (HUVEC) and (**C**) epithelial (OKF6) cells. CytoHubba with maximal clique centrality (MCC) analysis showing hub genes in (**B**) human endothelial (HUVEC) and (**D**) epithelial (OKF6) cells.

We identified *SNRPE*, *DKC1*, *BMS1*, *MRPS7*, *BIRC2*, *NFKB2*, *NFKBIZ*, and *STAT3* as important hub genes in endothelial cells and *RRP1B*, *RRS1*, *UTP14A*, *NOL6*, *SURF6*, *HK2*, *LDHA*, and *PFKFB3* as important hub genes in epithelial cells. Our PPI analysis identified eight hub genes (*STAT3*, *SNRPE*, *BIRC2*, *NFKB2*, *RRS1*, *SURF6*, *HK2*, and *LDHA*) from epithelial and endothelial cells based on MCC score cut-off (Figure 5).

## 3. Discussion

*Candida albicans* is a human opportunistic fungus that can cause life-threatening systemic infections, especially in immunocompromised patients. To better understand the host response to *C. albicans*, we investigated human endothelial and epithelial cell transcriptomics during *C. albicans* infection using consensus WGCNA.

Our results showed that the topmost enriched pathways in endothelial cells during *C. albicans* infection were TNF (tumor necrosis factor), AGE-RAGE signaling, NF-κB signaling, MAPK signaling, lipid, and atherosclerosis. These pathways are critical in regulating immune responses and are likely activated in response to *C. albicans* infection [26,27,28,29,30,31,32]. The endothelial cells, which are vital for maintaining vascular integrity, appear to have unique mechanisms for combating *C. albicans* infections [33,34].

The TNF pathway is involved in the regulation of cell survival, cell death, inflammation, and immune response [14,35,36]. It has been shown to play a role in the defense against *C. albicans* infection by inducing cytokine production (e.g., IL-6 and IL-8) and activating immune cells [37,38]. TNF signaling also stimulates the production of adhesion molecules, such as intercellular adhesion molecule-1 (ICAM-1) and vascular cell adhesion molecule-1 (VCAM-1), which allow immune cells to adhere to and traverse the endothelial barrier to reach the site of infection [14]. The AGE-RAGE signaling pathway has been implicated in regulating inflammation and cellular metabolism [39,40], and its activation enhances the host response to *C. albicans* infection in transplant patients [41,42,43]. Therefore, it contributes to various complications in transplant patients, including tissue damage, organ failure, and an increased risk of infection [41]. The NF-κB signaling pathway helps regulate immune responses [44] and inflammation and plays a critical role in the defense against *C. albicans* [28,45,46]. It is activated in response to *C. albicans* infection and leads to the production of a variety of cytokines, chemokines, and other immune mediators [45]. These molecules act to recruit immune cells to the site of infection and activate them, leading to the clearance of the pathogen [44]. The MAPK signaling pathway regulates cellular processes such as differentiation, proliferation, and survival [13,47], and it is activated in response to a variety of stimuli, including cytokines and growth factors, and leads to the activation of a range of downstream effector molecules during invasion of *C. albicans* [30,37]. These molecules play important roles in regulating cellular proliferation, differentiation, and apoptosis, and can also play important roles in regulating the immune response to infection [47]. Enriching lipid [48,49,50] and atherosclerosis [51,52,53] pathways may result from endothelial dysfunction during *C. albicans* infection. Dysregulation of lipid metabolism has been linked to a wide range of disease states, including atherosclerosis, diabetes, and obesity [48,49,50]. While atherosclerosis is typically associated with cardiovascular disease, recent studies have suggested that it may also play a role in other diseases, including infectious diseases [53].

In contrast, the enriched pathways in epithelial cells during *C. albicans* infection were glycolysis, pyruvate metabolism, amino acid, fructose, mannose, and vitamin B6 metabolism. These pathways suggest that epithelial cells may utilize metabolic pathways to generate the energy required for the immune response against *C. albicans*. These findings are particularly intriguing, as the epithelial cells, which line the surfaces of the body, play an essential role in the first line of defense against invading pathogens.

The glycolysis pathway has been known to play a critical role in energy production and is also important for immune cell function in *C. albicans* [54,55]. Similarly, pyruvate metabolism is important for cellular energy production and the regulation of cellular processes [54,56]. The upregulation of both glycolysis, fructose, mannose, and pyruvate metabolism in epithelial cells during *C. albicans* infection suggests that these cells are actively engaged in energy production to fuel their response to the infection. In *C. albicans*, amino acid metabolism aids in regulating immune responses [54,57]. The upregulation of amino acid metabolism in epithelial cells during *C. albicans* infection suggests that these cells actively synthesize and degrade proteins as part of their response to the infection. Vitamin B6 is a coenzyme that plays a critical role in amino acid metabolism, neurotransmitter synthesis, and immune function [58,59]. The upregulation of vitamin B6 metabolism in epithelial cells during *C. albicans* infection suggests that these cells may be actively utilizing this coenzyme to support their response to the infection. The enrichment of these metabolic pathways in epithelial cells may be a response to the energy demands of the immune response against *C. albicans*.

Furthermore, we found that mitophagy, necroptosis, and apoptotic processes, as well as hypoxia and HIF-1 signaling, were enriched in both endothelial and epithelial cells during *C. albicans* infection. These pathways play crucial roles in limiting the spread of the pathogen and promoting tissue repair.

Mitophagy is the selective degradation of damaged mitochondria and has been shown to play a role in regulating immune responses in *Candida* species [60,61]. The upregulation of mitophagy during infection suggests that epithelial cells actively eliminate mitochondria that have been compromised by the infection. This process is critical for maintaining cellular homeostasis and promoting cell survival. Necroptosis and apoptosis are a form of programmed cell death that is induced by inflammation and are important for the regulation of immune responses in the defense against *C. albicans* infection [62,63,64,65]. The upregulation of these pathways in epithelial cells during *C. albicans* infection suggests that these cells may be undergoing a controlled form of cell death as part of their response to the infection. This may help to limit the spread of the infection and promote the clearance of infected cells. In solid organ transplant patients, the immune response to *C. albicans* infection can be further complicated by immunosuppressive drugs, impairing the host’s ability to control the infection [22,23,24]. In these patients, necroptosis and apoptosis have been shown to contribute to the development of transplant-related morbidity and mortality and the development of allograft rejection [66,67,68,69,70]. HIF-1 signaling, a pathway activated by hypoxia, is crucial in regulating cellular responses [71,72,73] to low oxygen levels and immunity [74,75]. Our study found that hypoxia- and HIF-signaling-related genes were significantly enriched in both endothelial and epithelial cells during *C. albicans* infection. This could indicate that hypoxia and HIF-1 signaling are important in the host response to *C. albicans* infection, potentially through the regulation of immune responses.

Our findings provide a more detailed understanding of the cellular responses to *C. albicans* infection in endothelial and epithelial cells, which could be useful for developing targeted therapies. However, our study also has some limitations. Firstly, it was based on in vitro experiments using cell lines, and the results may not fully reflect the complexity of the in vivo environment. Secondly, our study is focused on transcriptomics data, and future studies using other omics approaches, such as proteomics and metabolomics, may provide a more comprehensive understanding of host–pathogen interactions. We were also limited to a single type of *Candida* species, and future studies should explore the transcriptomic changes in host cells during infection with other fungal species. Finally, our study was based on consensus WGCNA, and future studies using other bioinformatics tools may provide additional insights into the molecular mechanisms underlying host–pathogen interactions.

## 4. Materials and Methods

### 4.1. Data Collection and Quality Control

**Figure 6:**
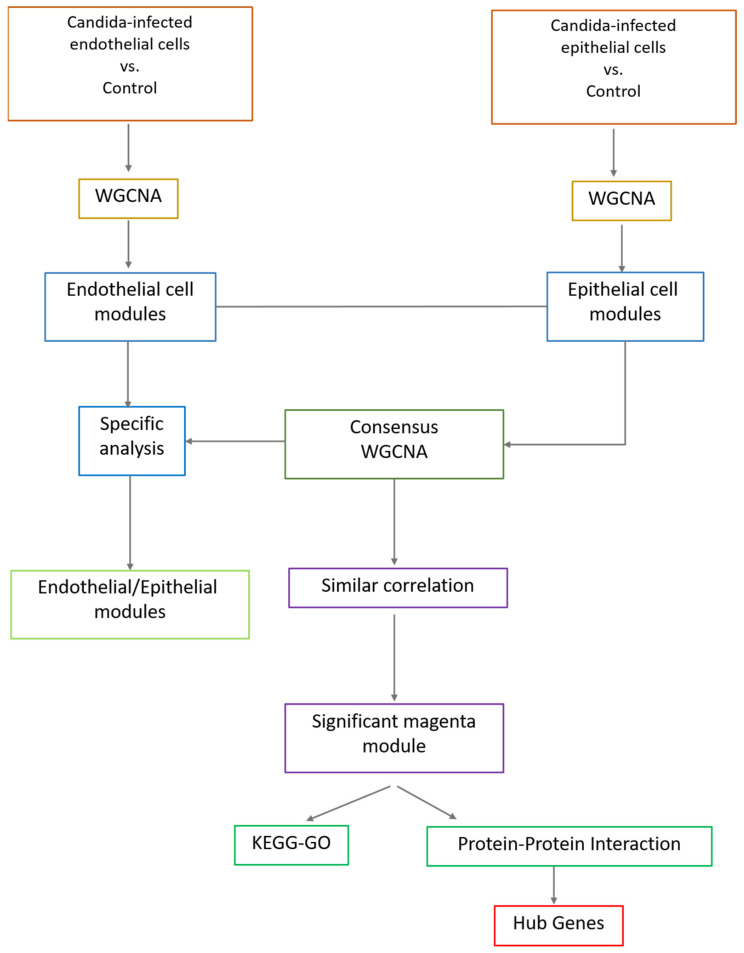
The overall methodology of the proposed study. The endothelial and epithelial cells were independently analyzed using WGCNA to retrieve the cell-specific modules. The consensus WGCNAs were performed to identify the similar and dissimilar modules. Finally, the downstream analyses were conducted using KEGG, GO, and PPI.

We utilized a publicly available dataset from NCBI Gene Expression Omnibus GSE56093 to explore the pathways related to *C. albicans* infection in epithelial and endothelial cells [9]. This dataset contains 88 samples and, from those, we used 32 samples of human epithelial and endothelial cells infected with *C. albicans* and their controls. The alignment of the raw sequences was completed by Liu et al., and the resultant processed reads per kilobase of transcript, per million mapped reads (RPKM), was used to perform normalization. We used the GCRMA limma package [76] for filtering out low read counts (i.e., less than 10 in 90% of the samples) and missing. The study outline is described in Figure 6.

### 4.2. Consensus Weighted Gene Correlation Network Analysis

We used the WGCNA package in R programming to build the correlation network [77]. The infection and control status was used as the clinical trait to identify the correlation. Firstly, two independent correlation networks were constructed for epithelial and endothelial cells [25]. We chose a soft threshold power of 8 and 15 for the human endothelial cells and oral epithelial cell samples, respectively, to calculate the correlations between the adjacent genes (Supplementary Figure S2). Subsequently, a consensus correlation network was built based on these modules using the consensus WGCNA pipeline [77]. We then calculated the consensus TOM and used this as an input for hierarchical clustering (Supplementary Figure S1). We identified modules using dynamic tree cut and further identified consensus module eigengenes (MEs) and clustered them based on consensus correlation across human endothelial and epithelial cell samples, then merged modules whose expression profiles were very similar. We identified significant modules by evaluating genes with high gene significance (GS) and module membership (MM) in human samples. We related the endothelial-specific modules to the consensus modules by Fisher’s exact test and investigated each pair’s overlaps by calculating the *p*-value. Lastly, intramodular connectivity was analyzed between human endothelial and epithelial cell modules using MTR > 0.35 and *p*-value < 0.05.

### 4.3. Functional Enrichment Analysis of Genes

To study the underlying functions and pathways associated with the significant module, we performed Gene Ontology. KEGG enrichment analyses were conducted using the enrichR package in R. The enrichment analyses were performed using a hypergeometric test, and Benjamini Hochberg multiple testing was used to correct the adjusted *p*-value. The cut-off for the adjusted *p*-value was set to 0.05 for KEGG and GO analyses. Significantly enriched terms are visualized with bar plots.

### 4.4. Protein–Protein Interaction

The consensus module genes were uploaded into the STRING database, and a high confidence interaction score ≥ 0.7 was used to reduce false-positive interactions. The resultant network output was loaded into Cytoscape. CytoHubba was used with the maximal clique centrality (MCC) algorithm to discover the hub genes in the PPI network.

## 5. Conclusions

In conclusion, our study provides insights into the differences and similarities between human endothelial and epithelial cell transcriptomics during *C. albicans* infection, highlighting the distinct responses of endothelial and epithelial cells to the pathogen. Endothelial cells appear to mount a response more focused on inflammatory and metabolic pathways, while epithelial cells rely heavily on metabolic pathways. Furthermore, our identification of hub genes and their potential interactions through PPI analysis provides a deeper understanding of the molecular networks involved in responding to *C. albicans* infection. Our findings suggest that the response of endothelial and epithelial cells to *C. albicans* infection may be influenced by the interplay between different signaling pathways, and future studies are needed to fully understand the complexity of host–pathogen interactions. Our findings may also have implications in developing targeted therapies for fungal infections and could facilitate future studies investigating the role of hub genes and pathways in other infectious diseases.

## Data Availability

All relevant R script files are available at https://github.com/surabhin15/ConsensusWGCNA (accessed on 7 May 2023).

## Supplementary Materials

The following supporting information can be downloaded at https://www.mdpi.com/article/10.3390/ijms241411748/s1.
