# Supplementary material for: Consensus Gene Network Analysis Identifies the Key Similarities and Differences in Endothelial and Epithelial Cell Dynamics after Candida albicans Infection
Source: Int J Mol Sci. 2023 Jul 21;24(14):11748. doi: 10.3390/ijms241411748 (PMC10380918; doi:10.3390/ijms241411748)
Supplement: Supplementary file 1 [file ijms-24-11748-s001.zip › Supplementary Figure S1.pdf]

A

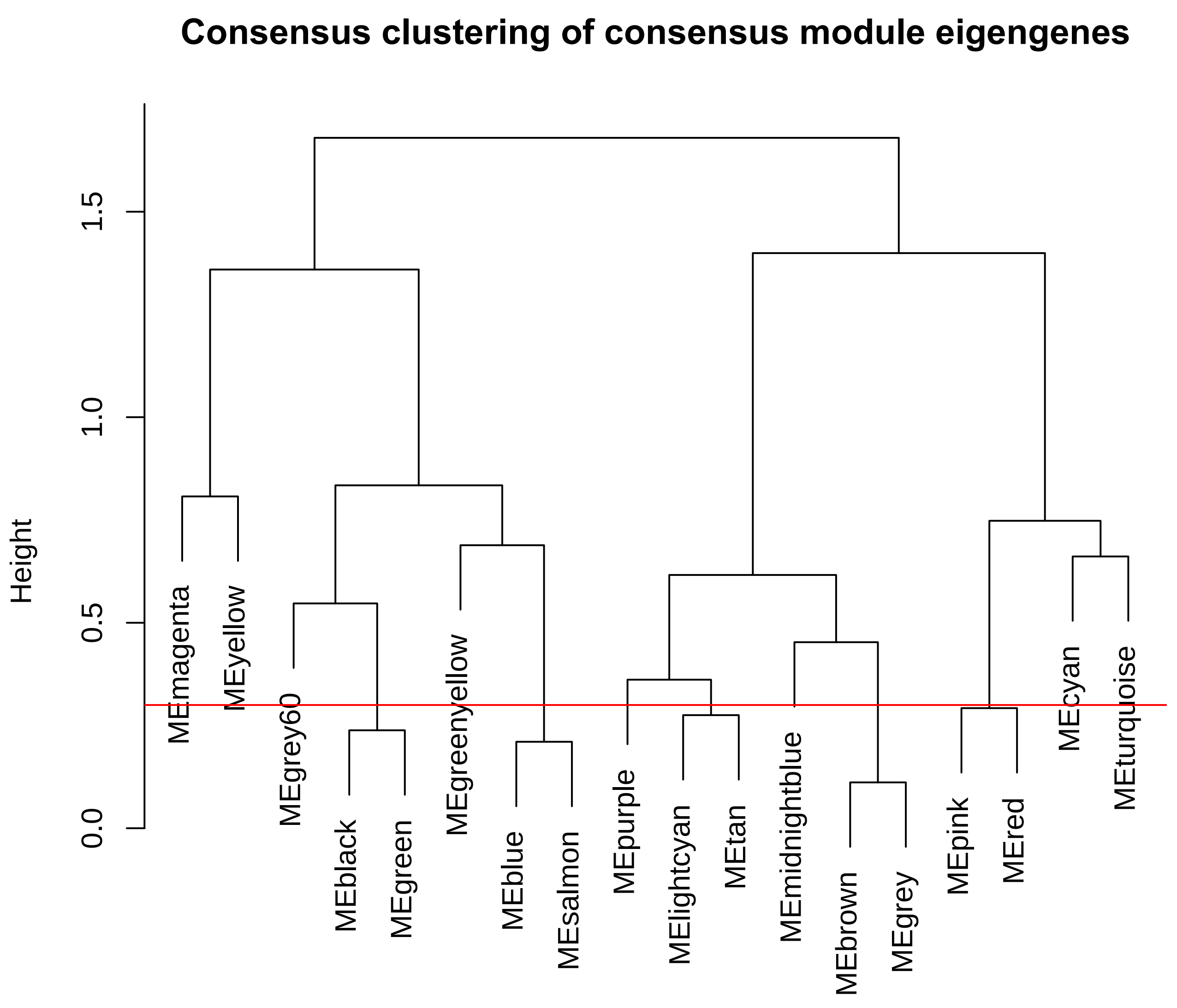
Consensus clustering of consensus module eigengenes
Height
0.0
0.5
1.0
1.5
MEmagenta
MEyellow
MEgrey60
MEblack
MEgreen
MEgreenyellow
MEblue
MEsalmon
MEpurple
MElightcyan
MEtan
MEmidnightblue
MEbrown
MEgrey
MEpink
MEred
MEcyan
MEturquoise

B

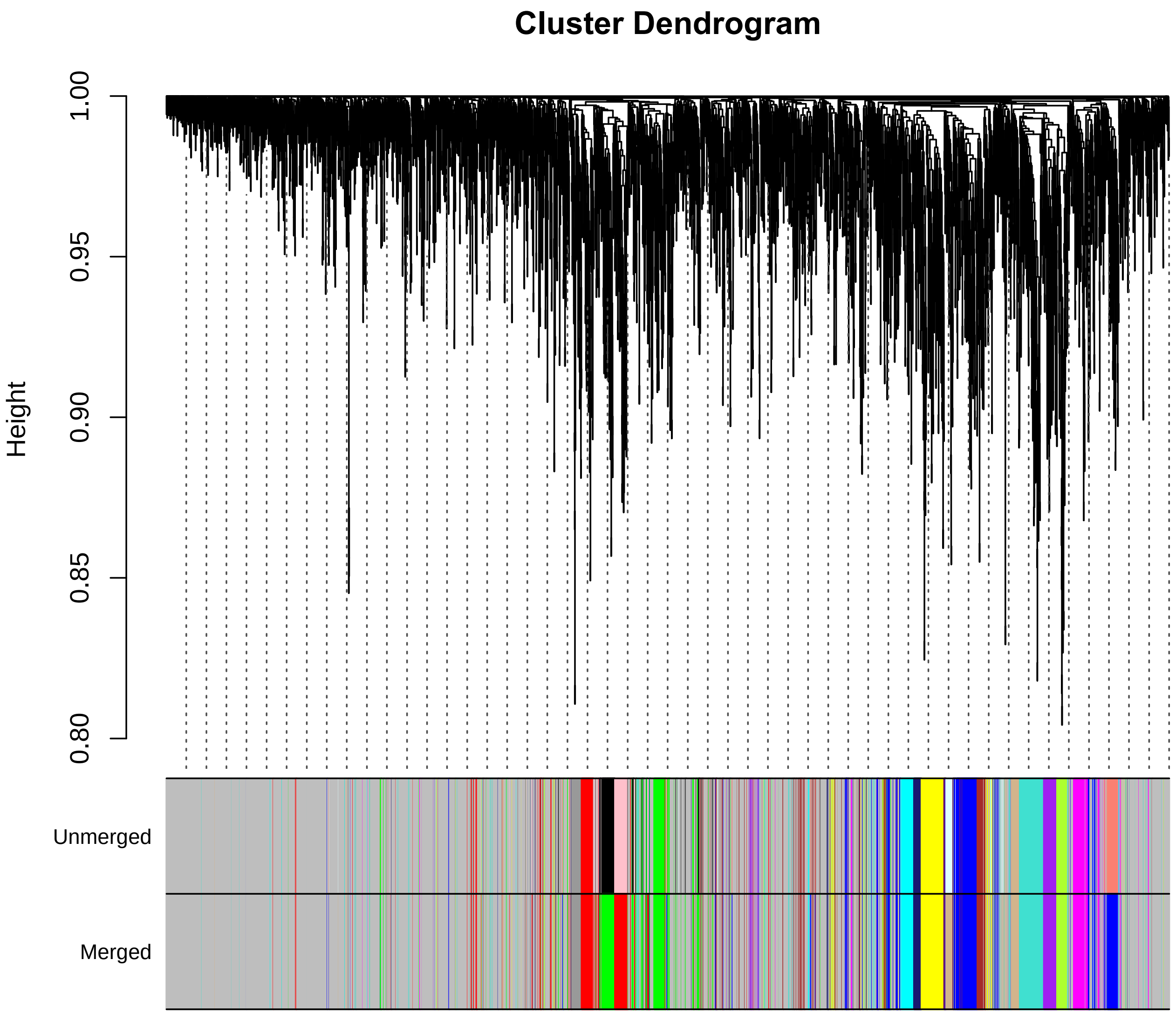

Supplementary Figure S1. Consensus Weighted Gene Correlation Network (A) clustering of module eigengenes, (B) The clustering of genes, unmerged modules, and merged modules
