# Supplementary material for: Consensus Gene Network Analysis Identifies the Key Similarities and Differences in Endothelial and Epithelial Cell Dynamics after Candida albicans Infection
Source: Int J Mol Sci. 2023 Jul 21;24(14):11748. doi: 10.3390/ijms241411748 (PMC10380918; doi:10.3390/ijms241411748)
Supplement: Supplementary file 1 [file ijms-24-11748-s001.zip › Supplementary Figure S2.pdf]

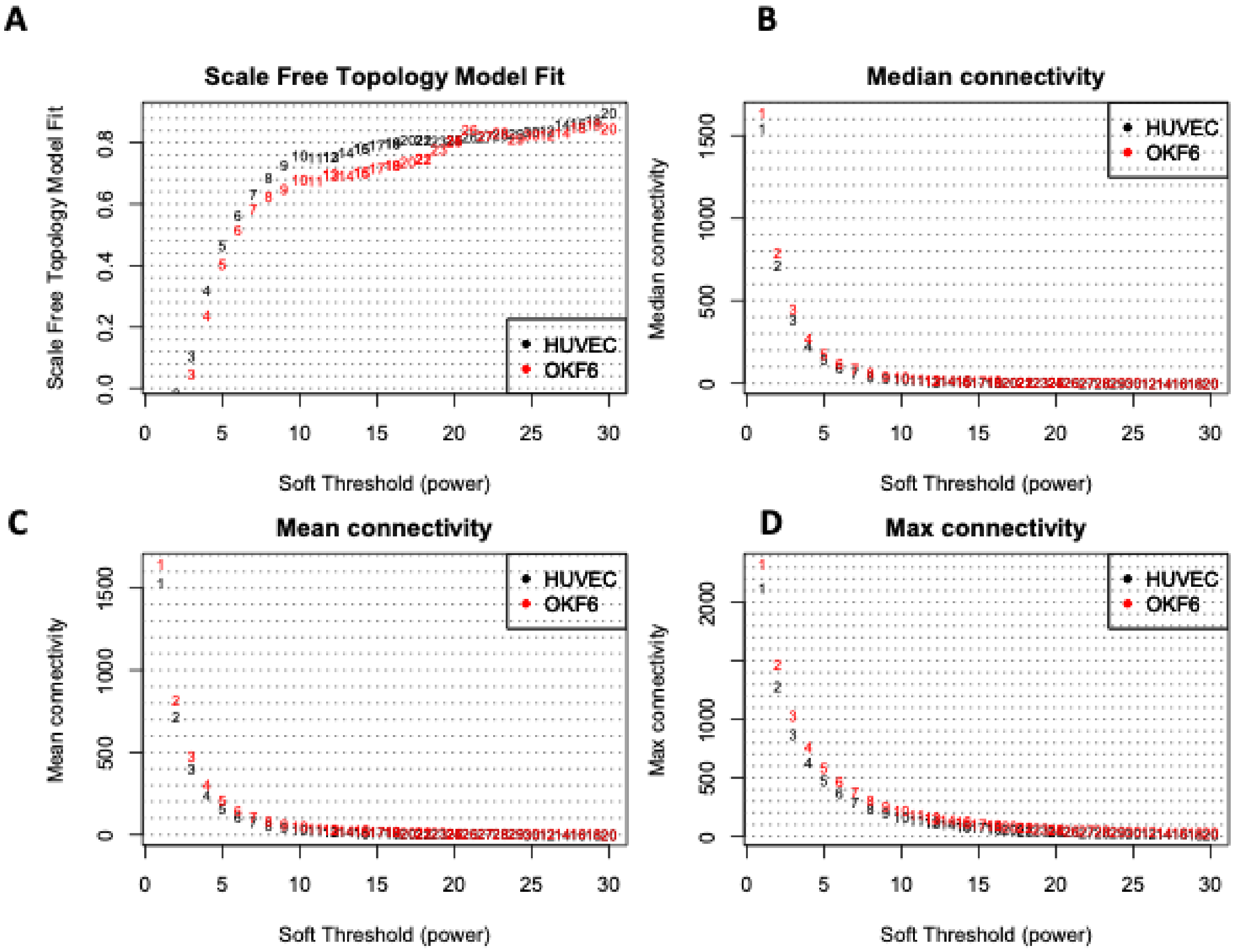

Supplementary Figure S2. Soft threshold power value (A) the scale-free topology model fit under different power; (B-D), the median, mean, and max connectivity (y-axis) drops with the increase of the soft-thresholding power.
